# A Paper-Based Near-Infrared Optical Biosensor for Quantitative Detection of Protease Activity Using Peptide-Encapsulated SWCNTs

**DOI:** 10.3390/s20185247

**Published:** 2020-09-14

**Authors:** Vlad Shumeiko, Yossi Paltiel, Gili Bisker, Zvi Hayouka, Oded Shoseyov

**Affiliations:** 1 Department of Plant Sciences and Genetics in Agriculture, Faculty of Agriculture, Food and Environment, The Hebrew University of Jerusalem, Rehovot 76100, Israel; vlad.shumeiko@mail.huji.ac.il; 2 Center for Nanoscience and Nanotechnology, Applied Physics Department, The Hebrew University of Jerusalem, Jerusalem 9190501, Israel; paltiel@mail.huji.ac.il; 3 Department of Biomedical Engineering, Faculty of Engineering, Tel Aviv University, Tel Aviv 6997801, Israel; bisker@tauex.tau.ac.il; 4 Institute of Biochemistry, Food Science and Nutrition, Faculty of Agriculture, Food and Environment, The Hebrew University of Jerusalem, Rehovot 76100, Israel

**Keywords:** optical biosensor, SWCNTs, protease, acute pancreatitis

## Abstract

A protease is an enzyme that catalyzes proteolysis of proteins into smaller polypeptides or single amino acids. As crucial elements in many biological processes, proteases have been shown to be informative biomarkers for several pathological conditions in humans, animals, and plants. Therefore, fast, reliable, and cost-effective protease biosensors suitable for point-of-care (POC) sensing may aid in diagnostics, treatment, and drug discovery for various diseases. This work presents an affordable and simple paper-based dipstick biosensor that utilizes peptide-encapsulated single-wall carbon nanotubes (SWCNTs) for protease detection. Upon enzymatic digestion of the peptide, a significant drop in the photoluminescence (PL) of the SWCNTs was detected. As the emitted PL is in the near-infrared region, the developed biosensor has a good signal to noise ratio in biological fluids. One of the diseases associated with abnormal protease activity is pancreatitis. In acute pancreatitis, trypsin concentration could reach up to 84 µg/mL in the urine. For proof of concept, we demonstrate the feasibility of the proposed biosensor for the detection of the abnormal levels of trypsin activity in urine samples.

## 1. Introduction

Disruption of enzymes’ homeostasis is associated with many pathological conditions. Upregulation or downregulation of enzyme expression is often used as a biomarker for disease diagnostics or as a drug target. A protease is an enzyme that hydrolyzes the amide bonds of proteins and breaks them into smaller polypeptides or single amino acids; thus, proteases play a crucial role in the maintenance of cells’ homeostasis. Proteases are classified into families based on their catalytic site, i.e., aspartic, cysteine, glutamic, metallo, serine, and threonine proteases [1]. They are involved in numerous intracellular and extracellular processes and are known to play an important role in cancer [2,3,4,5], cardiovascular disease [6,7], pancreatitis [8,9], diabetes [10], viral infections [11] (including the novel COVID-19 [12]), and microbial [13,14] infections. Almost 6% of the proteins in the human genome are proteases [15], and they can be found in biological fluids like saliva [16], serum [17], and urine [18,19]. Therefore, monitoring protease activity could aid in drug development, diagnostics, and treatment of various pathological conditions.

Biosensors can serve as compact, reliable, cost-effective, and easy-to-operate devices for point of care (POC) diagnostics. Depending on the method of the signal transducer, biosensors can be classified into five main categories: electrochemical, optical, piezoelectric, thermometric, and magnetic [20]. Optical biosensors are usually simpler in their configuration, maintenance, and handling compared to other types of biosensors [21]. The sensors themselves are physically separated from the detector, thus simplifying their replacement and preventing possible contamination of the device by biological samples. The detector itself, in many cases, could be a simple complementary metal oxide semiconductor (CMOS) camera—sometimes one that is integrated into a smartphone [22,23], thus significantly reducing the device cost. Optical biosensors that emit signals in the near-infrared (NIR) region are of particular interest in the biomedical field, as NIR wavelengths are less absorbed and scattered in biological tissues compared to visible wavelengths [24].

Semiconducting single-wall carbon nanotubes (SWCNTs) are one of the most promising nanoscale molecules for NIR photoluminescence (PL) and electrochemical-based sensing [25,26,27,28]. SWCNTs are characterized by two chiral indexes (n, m) according to their lattice structure [29]. NIR PL emission of SWCNTs depends on their chirality and falls between 900 and 1600 nm [9]. (6,5) chirality emits PL between 950 and 1100 nm [30], which enables creation of low-cost devices, as PL at around 1000 nm can be measured with the help of affordable silicon detectors instead of expensive and more complicated indium gallium arsenide (InGaAs) detectors [31,32].

It was demonstrated previously that SWCNTs can be dispersed in water using peptides [33], DNA [34], RNA [35], or various surfactants [36,37]. SWCNTs’ PL and conductivity were shown to be strongly influenced by changes in the local environment [38,39]. This exciting feature led to the development of various sensors. For example, SWCNT-based biosensors were developed for detection of small molecules [32,40,41], proteins [28,42,43,44], bacteria [45,46], ions [47], DNA [48,49], and RNA [50].

Trypsin protease is an extremely important serine protease found in the digestive system of humans and many other vertebrates [51]. It exclusively cleaves the C’-terminal of arginine and lysine residues [52]. Changes in trypsin activity are linked to pancreatic cancer [53] and pancreatitis [54,55]. It was reported that trypsin levels in serum and urine are dramatically increased during acute pancreatitis. While the normal range of trypsin concentration in the serum of a healthy person is 0.25 ± 0.1 μg/mL [56] and in the urine is about 115 to 350 ng/mL, patients with pancreatitis show maximum levels of 1.4 ± 0.6 [57] and 84.4  µg/mL, respectively [58]. One of the most frequently used methods for quantitative detection of proteins is enzyme-linked immunosorbent assay (ELISA) [59]. However, since proteases have catalytic activity, there is an interest in the quantification of enzyme activity and not only in its concentration [60,61,62]. The development of peptide-based assays to determine protease activity has advantages over conventional, non-specific protease assays using casein [63]. The success of the approach using magnetic nanoparticles [60], electrochemical sensing [64,65], calorimetry [66], mass spectrometry [67], quantum dots [68], and others was demonstrated for various proteases.

The current work presents a biosensor based on peptide-encapsulated (6,5) SWCNTs for the detection and quantification of protease activity. Detection of the active trypsin in urine samples was used as a model to demonstrate the capacities of the biosensor.

## 2. Materials and Methods

### 2.1. Chemicals and Reagents

*N*,*N*-diisopropylethylamine (DIEA)*, N*,*N*-dimethylformamide (DMF), diethyl ether, Oxyma Pure, *N,N*-Diisopropylcarbodiimide (DIC), and trifluoroacetic acid (TFA) were purchased from Biolab (Jerusalem, Israel). Fmoc-AA-OH, 2-(1H-benzotriazole-1-yl)-1,1,3,3-tetramethyluronium hexafluorophosphate (HBTU), and rink amide resin (0.53 mmol/g) were purchased from Chem-Impex (Wood Dale, IL, USA). SWCNTs (CoMoCAT™ Signis^®^ SG65), sodium cholate (SC), Bovine Serum Albumin (BSA) and poly (vinyl alcohol) were purchased from Sigma (Rehovot, Israel). Phosphate-buffered saline was purchased from Biological Industries (Beit HaEmek, Israel).

### 2.2. Peptide Synthesis

The HexCoil-Ala peptide developed by Grigoryan et al. [69,70] was synthesized with the CEM Liberty Blue™ Automated Microwave Peptide Synthesizer [71], on a 0.10 mmol scale, using rink amide resin (Chem Impex International Incorporated, Wood Dale, IL, USA), according to the manufacturer’s instructions. Briefly, 10% *w/v* piperidine was used as the Fmoc-deblocking reagent. Coupling was achieved using a 4-fold excess of Fmoc-AA-OH (0.2 M in DMF), DIC, and Oxyma Pure. The peptide was cleaved from the resin by a 3 h incubation in a 95% (*v/v*) TFA, 2.5% (*v/v*) in double-distilled water (DDW), and 2.5% (*v/v*) triisopropylsilane solution. The peptide was then precipitated by the addition of ether. After centrifugation (4 °C, 5000 RCF), the ether was aspirated, the pellet was frozen in liquid nitrogen, and lyophilized.

At random, peptides YK [(Y)_0.5_-(K)_0.5_]_20_ and WFK [(W)_0.33_-(F) _0.33_-(K)_0.33_]_20_ were synthesized as previously described [72,73], using a MARS VI multimode microwave. Briefly, random peptide synthesis results in a mixture that contains up to 2^20^ YK or 3^20^ WFK peptides. Coupling reactions were conducted with binary combinations of L-Fmoc-protected amino acids. Before coupling, an aliquot containing four equivalents (100 μmol) of the 1:1 amino acid mixture was activated in DMF with four equivalents of HBTU and eight equivalents of DIEA. The activated amino acid solution was then added to the solid-phase synthesis resin, and the reaction mixture was heated to 70 °C in a MARS VI multimode microwave (2-min ramp to 70 °C, 4-min hold at 70 °C), with stirring. Deprotection was achieved by adding 20% piperidine in DMF and heating (2-min ramp to 80 °C, 3-min hold at 80 °C), with stirring. After each cycle, the resin was washed three times with DMF.

The synthesis was validated by MALDI-TOF mass spectrometry.

Peptides restriction sites were predicted using an ExPASy PeptideCutter tool [74].

### 2.3. Preparation of Peptide-Encapsulated SWCNTs

Three peptide/SWCNTs and poly(vinyl alcohol)/SWCNTs (PVA/SWCNTs) suspensions were prepared using a protocol published elsewhere, with minor modifications [33]. Briefly, individual peptides and SWCNTs were mixed (1:1 mass ratio) in distilled water (DW) using a one-eighth-inch probe-tip sonicator, at 10 W, for 20 min. For the suspension of SWCNTs with PVA, SWCNTs were first dispersed in sodium cholate (SC), by sonicating SWCNTs in a 2 *w/v*% SC suspension at 10 W, for 1 h. The resulting solution was centrifuged twice for 40 min at 16,000× *g*, and the pellet was removed each time. PVA was added to a final concentration of 2%. The resulting suspension was dialyzed against DW for 24 h.

The SWCNTs’ concentration was calculated based on its absorbance at 632 nm, using the Beer-Lambert law with the extinction coefficient, ε_632_ = 0.036 L mg^−1^ cm^−1^ [75].

### 2.4. Sensor Preparation

**Figure 1:**
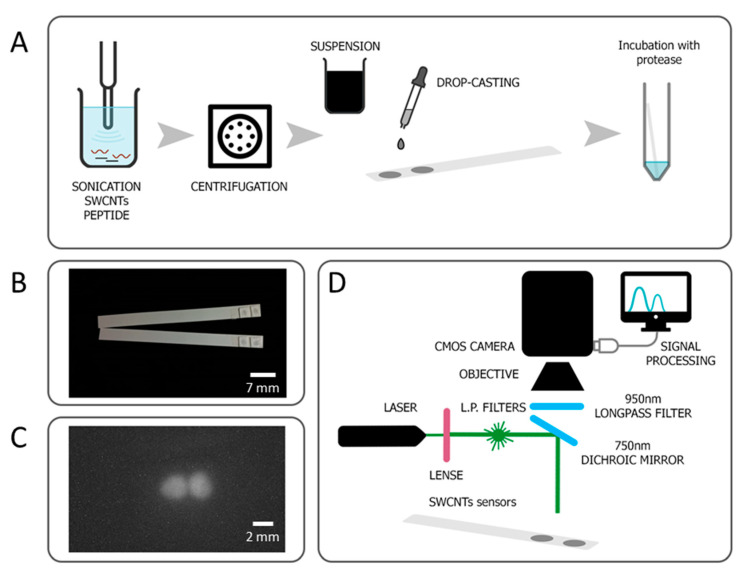
Developed sensor for trypsin detection. (**A**) Sensor preparation scheme, where two replicates of the same sensor are deposited on the same strip. (**B**) Photo of the prepared sensors strips. (**C**) Photoluminescence of the sensors, as captured with a CMOS camera. (**D**) Scheme of the optical setup for signal detection. Not to scale.

SWCNTs/peptide or SWCNTs/PVA suspensions (25 mg/L) were drop-casted (0.5 μL) on Whatman GF/C Glass Fiber paper (TAMAR), which was then dried at 37 °C, for 25 min. Drying was performed on an aluminum block in a closed oven with ventilation. The paper was cut into cubes around the sensors, and two repeating sensors were placed side by side on the dipstick with the help of double-sided tape (Figure 1A,B).

### 2.5. Protease Sensing

A custom setup was constructed for SWCNTs’ PL measurements (Figure 1D). PL emission of the peptide-encapsulated SWCNTs sensors was recorded using a XIMEA CMOS camera with 900 nm long-pass filters (Thorlabs), and a 532 nm laser (100 mW output, PGL-V-H-532 CNI) was used for excitation. Figure 1C displays photoluminescence of the sensors fixed on a dipstick, as captured with a CMOS camera. The PL intensity was measured before and after incubation with trypsin using Fiji ImageJ distribution [76]. 3D PL profiles were analyzed using the Interactive 3D Surface Plot plugin [77].

Informed written consent was obtained from a healthy volunteer who provided urine specimens. Urine samples were diluted in phosphate-buffered saline (PBS) 1:12 before the assay. The final PBS concentration was 1×, pH 7.5.

Trypsin (Biological Industries, Beit HaEmek, Israel) 1 mg/mL stock was prepared from powder and diluted to 1, 5, or 20 μg/mL immediately before the assay. In the protease inhibitor assay, Soybean Trypsin Inhibitor (SBTI) 50× (Biological Industries, Beit HaEmek, Israel) was diluted 1:50 and added together with trypsin. Each dipstick was placed in a separate 15 mL tube containing a 3 mL sample, inside an incubator, under 37 °C. To observe kinetics, the dipstick was taken out, analyzed, and placed back into the incubator. To evaluate the effect of urea (U5378, SIGMA) on the sensors PL, 10 or 20 mg/mL urea was added to the PBS and incubated for 3 h.

Statistical analysis was performed using GraphPad Prism version 6 (GraphPad Software, Inc., San Diego, CA, USA) for Windows.

## 3. Experimental Results and Discussion

**Figure 2:**
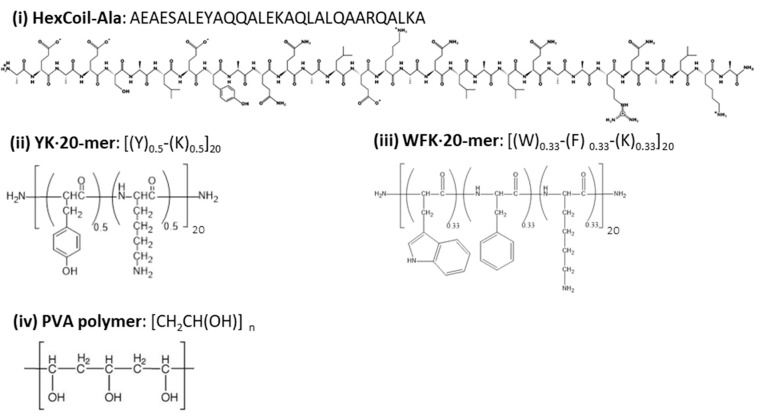
The structures of the three peptides and poly(vinyl alcohol) (PVA) polymer that were used for single-wall carbon nanotube (SWCNT) dispersion.

SWCNTs are extremely hydrophobic; thus, while they can be dispersed in organic solvents [78], they form insoluble aggregates in water. Aggregates diminish fluorescence [79] and do not enable interaction with proteins under physiological conditions. Covalent modification of SWCNTs may prevent aggregation but diminishes their optical properties [80]; thus, noncovalent conjugation is essential to create effective dispersion while preserving optical properties. Here, HexCoil-Ala [69], YK, and WFK peptides were used for noncovalent modification of SWCNTs (Figure 2). A PVA polymer was used as a control. YK and WFK peptides are rich in aromatic amino acids, which have been shown to exhibit remarkable affinity to SWCNTs [80]. Hydrophobic forces between the α-helical peptides and the sidewall of SWCNTs also enable their dispersion in water, and a number of possible peptide–SWCNT conformations were proposed [70,81]. Thus, a HexCoil-Ala was chosen as a feasible alternative to aromatic peptides.

**Figure 3:**
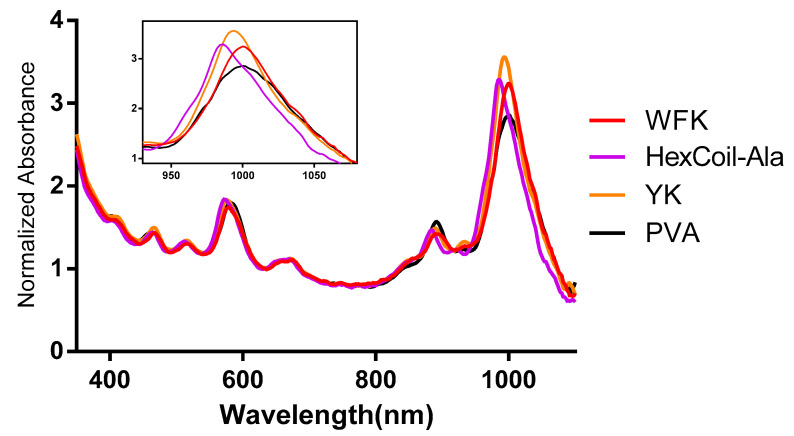
UV–visible absorption spectra of WFK, YK, HexCoil-Ala, and PVA-encapsulated (6,5) SWCNTs. The insert presents the 950–1050 nm part of the spectrum.

YK and WFK were synthesized as a random mixture of peptides. The previously reported technique [7,8,82] involves the incorporation of a mixture of amino acids in a defined proportion at each coupling step. This approach leads to a vast number of different peptides, a mixture that contains up to 2^20^ (for two types of amino acids) or 3^20^ (for three types of amino acids), 20 mer-long sequences. This technique is expected to enhance the chances of a sensitive and robust biosensor response by increasing the variability of the peptides. Moreover, random peptide mixtures are easier and cheaper to synthesize than specific sequence peptides, while still enabling reproducibility [73]. All three peptides have trypsin restriction sites, while PVA served as a negative control that was not cleaved by trypsin. All four molecules encapsulated SWCNTs successfully and created a stable dispersion in DW. The UV–VIS absorption spectrum (Figure 3) showed peaks at a wavelength of ~570 and ~987 nm that indicates excitonic optical absorption bands S_22_ and S_11,_ respectively [83], pointing out semiconducting (6,5) chirality-enriched SWCNTs. The slight differences in the absorption spectrum between different modifications could indicate dispersion quality.

One of the most useful methods used to transfer a laboratory-scale assay to a user-friendly kit, suitable for long-term storage, involves drying and fixing the sensing element to a paper, with well-known examples including home pregnancy tests and pH strips. Moreover, fixing sensors on the surface potentially prevents aggregation of SWCNTs upon reaction with buffer, trypsin, or urine. Therefore, SWCNTs suspensions were drop-cast on Whatman paper, dried in the oven at 37 °C, and then fixed on plastic strips (Figure 1A,B).

A custom optical setup was constructed; a schematic representation can be found in Figure 1D. As SWCNTs emit PL in the NIR region, an InGaAs-based detector is usually used for signal detection. Apart from the high costs of such devices, the InGaAs camera also has a lower resolution than silicon-based detectors. Choosing the (6,5) chirality of the SWCNTs, which has an emission peak at around 1000 nm, enabled use of a camera equipped with CMOS detector [32], which both boosts resolution and reduces costs. For (6,5) SWCNT excitation, we used a 532 nm laser [84]. Emission light was passed through a 900 nm long-pass filter and was captured by a 1.3 MP CMOS detector.

**Figure 4:**
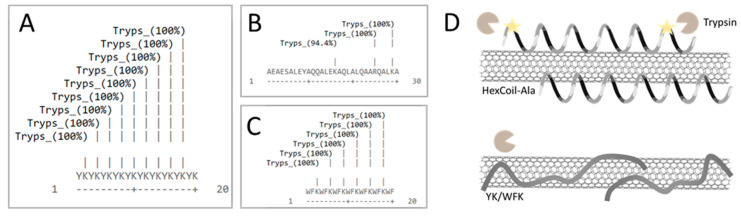
A map of the trypsin restriction sites on (**A**) YK, (**B**) HexCoil-Ala, and (**C**) WFK. (**D**) Schematic presentation of possible mechanisms that could account for the lack of trypsin reaction of YK and WFK sensors.

**Figure 5:**
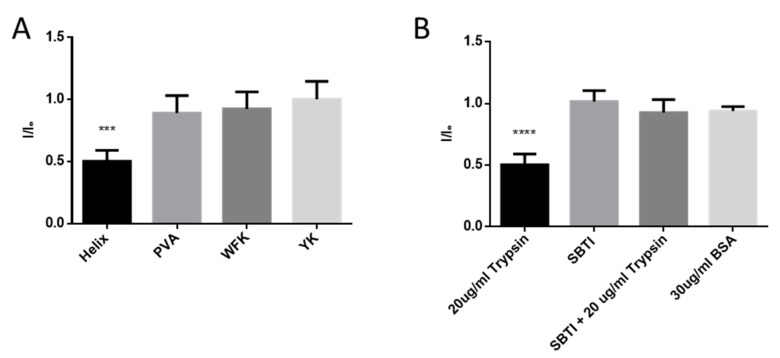
Trypsin effect on the sensors PL. (**A**) The PL levels of four different SWCNTs sensors after incubation with 20 μg/mL trypsin for 3 h as compared to initial PL value (*p* < 0.05, *n* = 5). (**B**) Comparing change in PL after incubation of the Helix sensor with 20 μg/mL trypsin for 3 h, in the presence or absence of trypsin inhibitor (SBTI). A total of 30 μg/mL of BSA served as a negative control. (One-way ANOVA, *p* < 0.05, *n* = 4.) Error bars represent standard deviation (SD).

Next, we tested the potential of the developed sensors to detect trypsin activity. All three peptides contain trypsin restriction sites; thus, a PL change was expected upon trypsin digestion. SWCNTs/PVA served as a negative control. The random YK peptide has nine trypsin restriction sites on average, WFK has six on average, and HexCoil-Ala peptide has three restriction sites (Defined) (Figure 4A–C). However, upon incubation with 30 μg/mL trypsin, only the HexCoil-Ala sensor showed a significant change in PL, while the two other peptide-encapsulated SWCNTs sensors together with the negative control (PVA/SWCNTs) showed no response (Figure 5A). A possible explanation for the lack of response of YK and WFK sensors despite the existence of the restriction sites is schematically illustrated in Figure 4D. While WFK and YK peptides are rich in amino acids with aromatic residues, the HelixCoil-Ala has none. Aromatic amino acids readily attach to the SWCNTs’ sidewall [80,85]. As they were randomly distributed throughout the peptide chain, aromatic amino acids may have induced a much flatter conformation close to the SWCNTs’ surface [86,87] as compared to the helix peptide. In contrast, a helical peptide likely preserves its 3D structure on the sidewalls of SWCNTs [69,70,81] and is thus much farther from the SWCNTs’ sidewall, enabling protease binding and peptide restriction. The change in peptide upon restriction leads to modulation of PL.

In order to verify that the PL change was caused by peptide restriction and not because of protein absorption on the SWCNTs’ surface, we tested an addition of the BSA protein alone and of the trypsin inhibitor together with trypsin. The addition of BSA or trypsin inhibitor (SBTI) alone led to no PL change in the HexCoil-Ala sensor, while the addition of SBTI together with trypsin fully prevented the drop in PL during a 3 h incubation (Figure 5B).

**Figure 6:**
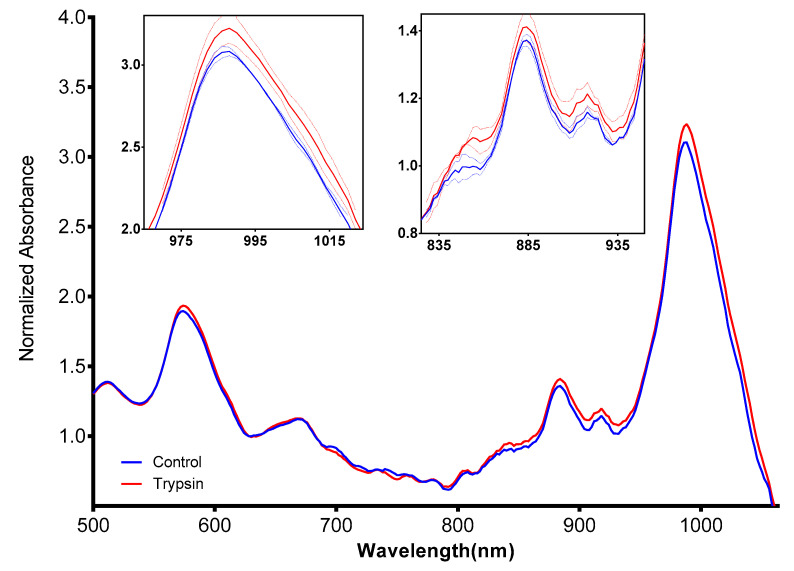
UV–Vis absorption spectra of HexCoil-Ala-encapsulated (6,5) SWCNTs before (blue) and after (red) incubation with 20 μg/mL trypsin. Left insert presents an enlarged 835–935 nm part of the spectrum, right insert the 975–1015 nm. Dashed lines represent SD, *n* = 4.

The absorption spectrum of SWCNTs depends, among other parameters, on their chirality [88] and on the encapsulating molecules [36]. The UV–Vis absorption spectrum of HexCoil-Ala/SWCNTs sensors following incubation with trypsin (Figure 6) indicated a change in the 590, 885, and 990 nm peaks. These data, together with PL decrease, clearly demonstrate changes in the wrapping molecule upon exposure to trypsin.

**Figure 7:**
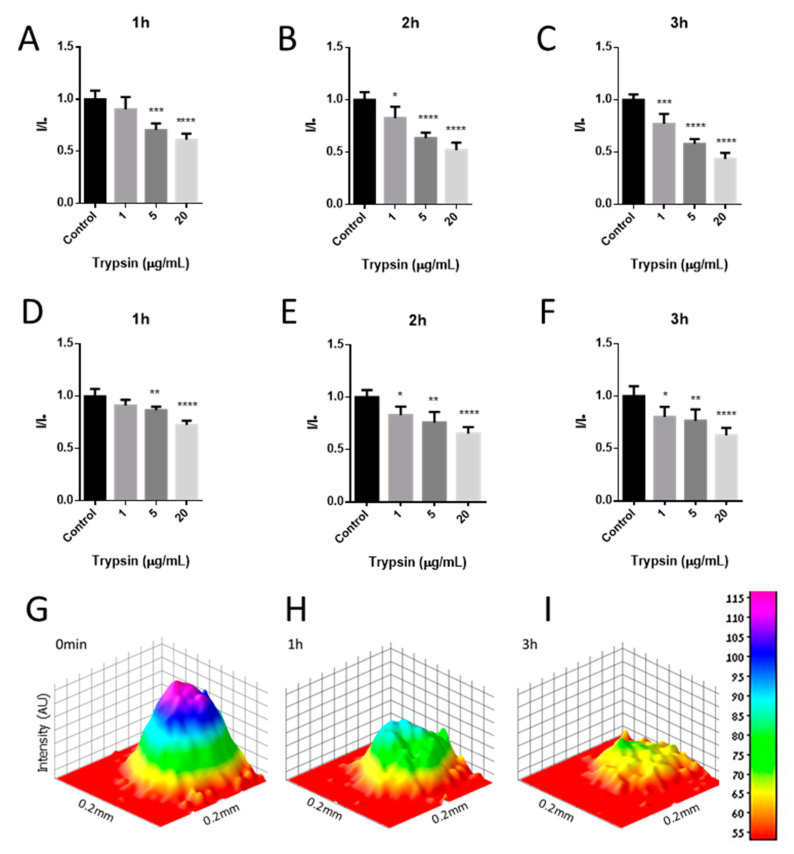
Time- and concentration-dependent changes in HexCoil-Ala-SWCNTs photoluminescence after incubation with trypsin or without trypsin (control); in (**A–C**) phosphate-buffered saline (PBS) or in 8.3% (**D–F**) urine. 3D photoluminescence intensity plot (**G**) immediately after addition, (**H**) 1 h after the addition, and (**I**) 3 h after the addition of 20 μg/mL trypsin. Error bars represent SD. One-way ANOVA, *p* < 0.05, *n* = 4.

Next, the sensitivity and kinetics of the sensor response to trypsin were evaluated (Figure 7A–C and Table 1), by measuring the PL every hour over the 3 h incubation period with 1, 5, or 20 µg/mL trypsin. After the first hour of incubation, 5 and 20 µg/mL trypsin induced a significant drop in HexCoil-Ala/SWCNTs’ PL, while 1 µg/mL trypsin did not cause a decrease in PL. However, after the second hour of incubation, 1 µg/mL trypsin also induced a significant decrease in PL signals, which further declined after 3 h of incubation. The 3D photoluminescence intensity plot (Figure 7G–I) presents PL distribution across the sensor geometry and displays a significant drop in PL in the middle of the dot, after incubation with trypsin. These results clearly show the dependence of SWCNTs’ PL both on the amount of trypsin and on the incubation time. Moreover, the intensity of PL changes correlated with trypsin concentrations, suggesting the proposed dipstick as a quantitative biosensor of trypsin activity under the tested conditions.

**Figure 8:**
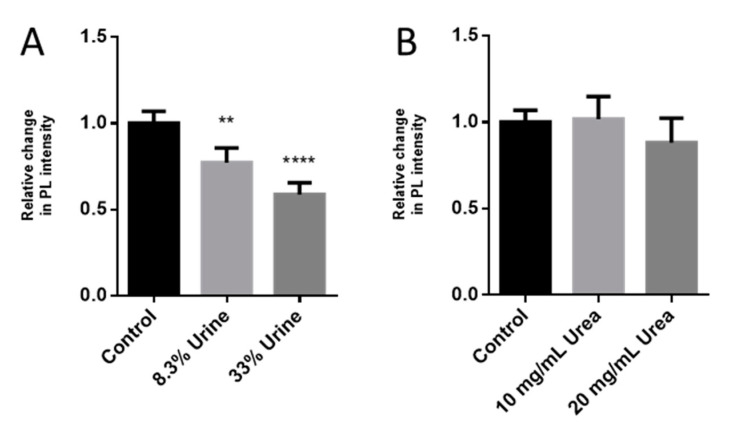
Effect of the urine and urea concentration on the sensors’ PL. Response of the biosensor to addition of (**A**) 8.3% or 33% urine and (**B**) 10 or 20 mg/mL urea. Error bars represent SD. One-way ANOVA, *p* < 0.05, *n* = 4.

To examine the option of detecting trypsin activity in a more complex environment, the same assay was performed in urine samples. During acute pancreatitis, trypsin concentration in urine could reach up to 84.4 µg/mL. We added different concentrations of trypsin (12, 70, and 1 mg/mL) to human urine. The samples were diluted 1:12 (8.3% *v/v*) in PBS before the assay to final trypsin concentrations of 1, 5, and 20 µg/mL, respectively. Similar to the tests with pure trypsin samples, the 1 µg/mL trypsin added to urine led to a significant decline in SWCNTs’ PL only after 2 h, while the higher concentrations of protease already induced PL changes during the first hour of the assay (Figure 7D–F). Overall, however, the relative decline in PL after incubation with trypsin was weaker in the presence of urine as compared to tests performed in PBS. Moreover, the absolute PL intensity was influenced by the concentration of the urine added to the reaction (Figure 8A). In the presence of 8.3% urine alone, without trypsin, the PL intensity declined by 33%, while the addition of 33% urine (1:3) caused almost a 50% drop in PL. One of the main components of urine is urea, with human urine containing about 9.3 g/L urea [89]. However, even at higher urea concentrations of 10 and 20 g/L, no significant effect on PL was noted (Figure 8B). Thus, some other urine component or components seemingly impact the PL of SWCNTs as well as reduce biosensor response to trypsin.

## 4. Conclusions

In summary, this work presented a disposable, paper-based NIR optical biosensor based on peptide-encapsulated (6,5) SWCNTs for trypsin activity detection. The biosensor response was recorded using a CMOS camera, thus potentially reducing the cost of the final device. Three different peptides were tested for SWCNTs’ modification, i.e., WFK, YK, and HexCoil-Ala. While all three peptides effectively dispersed SWCNTs in an aqueous solution, only HexCoil-Ala was responsive to trypsin. The developed biosensor was able to detect activity of 1–20 μg/mL trypsin after a 2 h incubation at 37 °C. The proposed biosensor also detected different concentrations of active trypsin in urine, a complex biological liquid with concentrations relative to acute pancreatitis. Although the urine affected both the baseline PL of SWCNTs and the extent of the fluorescence response, we have demonstrated that our biosensor can detect trypsin and differentiate between trypsin concentrations. Recent studies have reported the development of trypsin biosensors with a limit of detection of 8.6 ng/mL using a quartz crystal microbalance (QCM) [59] and 60 ng/mL using interferometric reflectance spectroscopy (IRS) [58] under the optimum conditions. Although the minimal tested trypsin concentration in this work was 1 μg/mL, we assume that after further careful optimization, the sensitivity could be significantly enhanced. For a single sensor, we used as little as 12.5 ng of SWCNTs, making this assay very cost-effective. Moreover, here, we present a biosensor based on a single-use disposable paper dipstick and an affordable CMOS camera. On the other hand, in most cases, both QCM and IRS sensors are based on expensive substrates and complicated equipment. In addition, our paper-based optical biosensor enables remote measurements, thus significantly reducing the risks for equipment contamination. The presented technique may be applied in the development of optical biosensors designed to detect other proteases in complex environments, e.g., urine, blood, or saliva.

## Figures and Tables

**Table 1:** A table representation of the relative intensity values ± SD of time- and concentration-dependent changes in HexCoil-Ala-SWCNTs’ photoluminescence after incubation with trypsin in PBS or in 8.3% *v/v* urine. *p* < 0.05, *n* = 4.

	Control	1 µg/mL	5 µg/mL	20 µg/mL
PBS				
1 h	1 ± 0.0845	0.905 ± 0.118	0.706 ± 0.0576	0.612 ± 0.0623
2 h	1 ± 0.0746	0.826 ± 0.108	0.636 ± 0.0709	0.521 ± 0.0502
3 h	1 ± 0.0512	0.770 ± 0.0952	0.580 ± 0.0577	0.435 ± 0.0456
8.3% Urine				
1 h	1 ± 0.0694	0.911 ± 0.0554	0.866 ± 0.0432	0.724 ± 0.0336
2 h	1 ± 0.0700	0.829 ± 0.0818	0.759 ± 0.0611	0.654 ± 0.101
3 h	1 ± 0.0953	0.802 ± 0.0965	0.766 ± 0.0705	0.627 ± 0.109
